# MCR-1 Gene Encoded Colistin-Resistant *Escherichia coli* in Raw Chicken Meat and Bean Sprouts in Malaysia

**DOI:** 10.1155/2020/8853582

**Published:** 2020-07-28

**Authors:** Erkihun Aklilu, Kausalya Raman

**Affiliations:** Faculty of Veterinary Medicine, Universiti Malaysia Kelantan, Locked Bag 36, Kota Bharu, Kelantan, Malaysia

## Abstract

This study was conducted to detect the presence of colistin-resistant *Escherichia coli* (*E*. *coli*) in raw chicken meat and bean sprouts collected from local markets and to determine the antimicrobial resistance patterns of the *E*. *coli* isolates. A total of 100 samples, comprised of 50 raw chicken meat and 50 bean sprouts, were collected and processed. Kirby-Bauer method was used to determine the antimicrobial resistance patterns, and PCR amplification was used to detect *E*. *coli* species-specific and colistin resistance (*mcr*-1 and *mcr*-2) genes. The results showed that 52.1% (12/23) of the *E*. *coli* isolated from raw chicken meat were positive for the colistin resistance encoding gene, *mcr*-1, whereas all the *E*. *coli* isolates from bean sprouts were negative for colistin resistance encoding genes. The findings show that chicken meat contaminated with colistin-resistant *E*. *coli* may pose public health risk to the consumers. Hence, prudent usage of antibiotics and hygienic handling of food items helps to prevent and combat the risks of spreading of colistin-resistant *E*. *coli* and the public health risks it may pose. More comprehensive and large-scale studies focusing on all the possible sources of colistin-resistant *E*. *coli* are recommended.

## 1. Introduction

Antimicrobial resistance (AMR) has been one of the alarming issues in recent decades. It has been a global threat drawing a global attention from several fields including medical science, veterinary, and agricultural fields. The rise and spread of AMR also led to enormous health and economic impacts. Though the constant warnings of the rise in antimicrobial resistance are not new, issues related to the impending threats of AMR were raised well before antimicrobials were established and used on a wider scale [1]. It did not take long before several pathogens developed resistance towards the available antimicrobials. Such incidences were fairly attributed to the rampant and irritational use of antimicrobial in human health, veterinary, and agricultural sectors. This in turn led to the emergence, evolution, and spread of numerous antibiotic-resistant bacteria.

Antimicrobial resistance in foodborne pathogens are of concern in recent days, especially when widely used in farms such as poultry production to control infectious diseases [2]. Hence, as antibiotic treatment persists, it will be one of the contributing factors to the emergence, selection, and spreading of antibiotic-resistant microorganisms in both veterinary and human medicine [3, 4]. As it has been reported by several studies, infection caused by drug-resistant bacteria are much more difficult to be treated with the commonly available antibiotics [5]. Documented investigations have showed that the use of antibiotics can lead to the emergence and dissemination of resistant *E*. *coli* which in turn could pose public health risks [2]. *Escherichia coli* is a facultative anaerobic bacteria found in gastrointestinal tract of humans and animals. Generally, *E*. *coli* is a harmless microbe; however, there are pathogenic strains of *E*. *coli* that may cause different diseases in humans and animals [6]. Foods of plant origin, particularly vegetables, are prone to contamination by different species of bacteria. Such contaminations may happen by the use of insufficiently treated and contaminated water and fertilizers or through other contaminants during cultivation. Moreover, animals may also acquire different pathogenic bacteria through water or food contamination by human and other animals' wastes. The unhygienic handling of meat during processing has also been known to cause contamination of meats by various pathogenic bacteria [6].

The use of polymyxins (colistin) has been revived in recent years after the drug was almost abandoned decades ago due to its potential side effects. The revival of colistin is due to the fact that it emerged as the drug of choice in the face of the emergence of multidrug-resistant Gram-negative bacteria and the lack of potent new antibiotics to treat infections caused by these pathogens [7]. Moreover, colistin has been widely used in animal production industry to enhance productivity and control diseases. These in turn led to the emergence of colistin resistance in *E*. *coli*, and recently the first plasmid-mediated colistin resistance determinants, *mcr*-1, *mcr*-2, *mcr*-3, *mcr*-4, and *mcr*-5, have been identified in Enterobacteriaceae, mostly in *E*. *coli* [8]. The continued discovery of different colistin resistance mechanisms and determinants of resistance raises a serious concern as colistin has been considered as a last-resort medication for infections caused by Gram-negative bacteria that are resistant to multiple antibiotics.

In Malaysia, the availability of data and reports on the prevalence and antibiotic resistance patterns of *E*. *coli* strains, particularly as to the occurrence of colistin resistance in *E*. *coli* and other Enterobacteriaceae, is still not well documented and reported. As such it is crucial to create awareness of the existence of the pathogen in the food chain and subsequently its public health implications [9]. Moreover, to the best of our knowledge, there has never been any report on the occurrence of colistin-resistant *E*. *coli* in foodstuffs (chicken and vegetables) in the country, and there are very few similar investigations conducted elsewhere. Therefore, this study was conducted to determine the occurrence of colistin-resistant *E*. *coli* raw chicken meat and bean sprouts from wet markets, supermarkets, and retail shops from local markets and to determine the antimicrobial resistance patterns of the *E*. *coli* isolates towards selected antimicrobial agents including colistin.

## 2. Materials and Methods

### 2.1. Sample Collection and Preparation

One hundred (*n* = 100) raw food samples comprised of fifty (*n* = 50) raw chicken meat samples and fifty (*n* = 50) bean sprouts were collected from supermarkets, markets, and road side stalls of different locations in Kota Bharu. Each sample was placed into Ziploc bags and 10 mL normal saline water was added into each sample collection bag. The samples were brought to Bacteriology Laboratory, Faculty of Veterinary Medicine, Universiti Malaysia Kelantan in ice box, and aseptic techniques were used through to reduce contamination. All the samples were processed and subsequently subjected to various bacteriological, biochemical, and molecular tests in the laboratory. Buffer Peptone Water (BPW) (Oxoid, UK) was used as the primary preenrichment broth to permit recovery of bacteria after sublethal injury caused by heat, preservatives, or other processing techniques. MacConkey agar (Oxoid, UK) was used to isolate Enterobacteriaceae family. Eosin Methylene Blue (EMB) agar (Oxoid, UK) was used as a differential and selective medium to screen for the growth of *E*. *coli*.

### 2.2. Isolation and Identification of *E*. *coli*

After collection, all samples were homogenised using stomacher and 1 mL of the suspension was transferred into 10 mL buffered peptone water using disposable pipette and the homogenates were incubated at 37°C for 16–18 h. After an overnight incubation in the preenrichment broth, a small amount of inoculum from BPW was transferred into MacConkey agar (Oxoid, UK) using a disposable pipette and streaked using a sterile wire loop. The inoculated plates samples were then incubated at 37°C for 24 h. After inoculation and isolating colonies from MacConkey agar, EMB (Oxoid, UK) was further used for screening. Greenish metallic sheen colonies on EMB were transferred to Nutrient Agar to nourish and maintain the colonies before conducting biochemical test for *E*. *coli*. Further biochemical tests and Gram's staining were conducted to identify *E*. *coli* phenotypically.

### 2.3. Antimicrobial Susceptibility Test

All the confirmed *E*. *coli* isolates were subjected to antimicrobial susceptibility towards colistin, amoxycillin, gentamycin, and enrofloxacin using Kirby-Bauer methods. The guidelines of Clinical and Laboratory Standard Institute (CLSI) was used as a reference for the determination of susceptibility [10]. Mueller–Hinton Agar (MHA) (Oxoid, UK) was used for antimicrobial sensitivity test, and a previously identified colistin-resistant *E*. *coli* from our research was used for a quality control. A swab of single colony from nutrient agar plate was transferred into test tube with 10 mL containing 0.9% normal saline. Then, the turbidity of the samples was compared with 0.5% of McFarland standard and the suspension was uniformly streaked on MHA. After placing the antibiotic discs aseptically, the plates were incubated at 37°C for 24 h and the zone of inhibition was measured and interpreted according to CLSI guidelines [10].

### 2.4. Detection of *E*. *coli* by Polymerase Chain Reaction (PCR)

#### 2.4.1. DNA Extraction and Primer Sequence

The genomic DNA was extracted using GF-1 Bacterial DNA Extraction Kit (Vivantis, Malaysia) following the manufacturer's recommendations. The primers used in this study for PCR identification of *E*. *coli* species and their resistance genes for colistin genes were selected based on previous studies. The PCR analysis was conducted for *E*. *coli* genes of family (*Pho*) with primers, Pho-F: 5′-GTGACAAAAGCCCGGACACCATAAATGC-3′ and Pho-R: 5′-TACACTGTCATTACGTTGCGGATTTGGCG-3′; colistin resistance genes *mcr*-1 (MCR-1F: 5′-CTCATGATGCAGCATACTTC-3′ and MCR-1R: 5′-CGAATGGAGTGTGCGGTG-3′) and *mcr*-2 (MCR-2F: 5′-TGTTGCTTGTGCCGATTGGA-3′ and MCR-2R: 5′-AGATGGTATTGTTGGTTGCTG-3′) [11–13]. Primers were obtained from Integrated DNA Technologies (Singapore).

### 2.5. Polymerase Chain Reaction (PCR) Amplification

#### 2.5.1. Amplification of *E*. *coli*-Specific Genes

As for the amplification procedure, the PCR mixtures were prepared for all samples that were phenotypically identified as *E*. *coli*. Two microlitres of bacterial DNA was amplified in 12.5 *µ*L of 2× Taq Master Mix (Vivantis, Malaysia) which consist of Taq DNA Polymerase (0.05 *µ*/*µ*L), 2× ViBuffer A, 0.4 Mm dNTPs and 3.0 Mm MgCl2, 8.5 *µ*L of nuclease-free water, and 1 *µ*L of each primer, respectively. PCR reaction for *E*. *coli* species-specific gene, the protocol was set as initial denaturation at 94°C for 2 minutes, final denaturation at 94°C for 1 minute, annealing at 56°C for 1 minute, extension at 72°C for 1 minute, cycle repeated at step 2 (94°C for 1 minute), followed by final extension at 72°C for 10 minutes and kept on hold at temperature 12°C.

### 2.6. Amplification of Colistin Resistance Encoding Genes

As for amplification of colistin resistance genes, 2 *µ*L of bacterial DNA was added into 12.5 *µ*L of 2× Taq Master Mix (Vivantis, Malaysia) which consist of Taq DNA Polymerase (0.05 *µ*/*µ*L), 2× (ViBuffer A, 0.4 Mm dNTPs and 3.0 Mm MgCl_2_), 8.5 *µ*L of nuclease-free water, and 1 *µ*L of each primer. The PCR protocol condition for MCR1F2/MCR1R2 primer pairs was with initial denaturation of DNA at 94°C for 3 minutes, followed by final denaturation at 94°C for 45 seconds, annealing at 60°C for 1 minute, and extension at 72°C for 3 minutes; then, the cycle was repeated 30 times at step 2 (94°C for 45 seconds), subsequently to final extension at 72°C for 4 minutes and finally, kept on hold at 4°C. Moreover, PCR protocol condition for MCR2IF/MCR2IR was carried out at initial denaturation of 94°C for 3 minutes, final denaturation at 94°C for 2 minutes, annealing at 65°C for 2 minutes, extension at 72°C for 3 minutes, for 30 cycles, then, final extension at 72°C for 4 minutes before holding at 4°C.

### 2.7. PCR Results Analysis

Analyses of the PCR products were conducted on 1.0% agarose gel (Agarose Vivantis, Malaysia) prepared in 60 mL of 1 × TBE Buffer added with 1.2 *µ*L of Midori Green (Nippon Genetics Europe, Germany) for *mcr*-1 gene. Meanwhile, as for *E*. *coli*, *mcr*-2 PCR products, it was analysed in 1.2% agarose gel prepared in 60 mL of 1 × TBE Buffer added with 1.2 *µ*L of Midori Green. Then, electrophoresis was conducted at 80 V, 400 A for 45 minutes in a gel electrophoresis system for *mcr*-1, on the other hand, at 100 V, 400 A for 40 minutes for *E*. *coli*, *mcr*-2. As for the final step, the gel was photographed and analysed using Gel Doc™ EZ Imager (Bio-Rad, USA).

## 3. Results

### 3.1. Isolation and Identification of *E*. *coli*

Based on the routine phenotypic isolation and identification, presumptive *E*. *coli* isolates were detected in 54% (27/50) of raw chicken meat and 20% (10/50) of the bean sprouts. Hence, the detection rate using phenotypic identification of *E*. *coli* in this study was 31% (31/100).

### 3.2. Antimicrobial Sensitivity Test

As for AST results, four antibiotics (amoxycillin/clavulanic acid, colistin, enrofloxacin, and gentamicin) were used, and based on the sensitivity of *E*. *coli* isolates from raw chicken meat, the highest number of samples showed resistance towards amoxycillin, followed with enrofloxacin, colistin, and gentamicin. Meanwhile, as for the bean sprouts, most of the resistance detected was against amoxycillin and colistin; however, no resistance towards enrofloxacin and gentamicin was detected. Moreover, MDR *E*. *coli* were also detected with resistance ranging from two to all antibiotics tested. The outcome of the determination of MDR isolates observed mostly in raw chicken meat as compared to bean sprouts with none of the isolates showing MDR. Tables 1 and 2 show the overall AST results and MDR status of the *E*. *coli* isolates from both food samples.

### 3.3. Molecular Identification

#### 3.3.1. PCR Results for *E*. *coli*-Specific *mcr*-1 and *mcr*-2 Genes Detection

Out of 31 presumptive *E*. *coli* isolates, 29 samples were positive for the presence of *E*. *coli* species-specific gene at 903 bp. Out of these 29 samples, 23 were from raw chicken meat and 6 were from bean sprouts. As for the detection of the resistance gene *mcr*-1, all the 29 samples were confirmed to be *E*. *coli* through PCR; 12 isolates were positive for resistance gene with the presence of amplification products at 1674 bp. All the 12 samples were from raw chicken meat and none of the isolates from bean sprouts samples showed the presence of the gene encoding for colistin resistance. Meanwhile, all the *E*. *coli* isolates from both food samples were negative for *mcr*-2 gene.

## 4. Discussions

Based on the results from routine bacterial culture and biochemical results, 46% (23/50) and 12% (6/50) of raw chicken meat and bean sprouts were, respectively, positive for *E*. *coli*. *E*. *coli* is commensally present in all warm-blooded animals at the enteric regions; hence, a high percentage of *E*. *coli* isolates to be obtained in raw chicken meat as compared to bean sprouts might be due to the higher probability of contamination of chicken meat by intestinal contents during processing and handling of chicken meat. Handling of meat and animal carcasses, cross contamination from soil, processing instruments, and the use of contaminated water for washing purpose can be major sources of contamination of chicken meat [14]. In addition to *E*. *coli*, other members of the Enterobacteriaceae family including *Enterobacter aerogenes* and *Klebsiella pneumoniae* were also identified in this research. Previous studies have reported that presence of *E*. *coli* in food is often considered as an indicator for the presence of other pathogenic bacteria in the respective food items [15]. As to the 24% detection rate of *E*. *coli* in bean sprouts in this study, the results may imply the high occurrence of contaminations of bean sprouts possibly through usage of contaminated water or untreated sewage water used as fertilizers that could possibly be mixed with manure of animals [15]. Several studies have reported foodborne pathogens isolated from agricultural products including vegetables and fruits. Likewise, these studies have also linked occurrence of food poisoning cases to contaminated vegetables and fruits [16, 17].

The AST results for *E*. *coli* isolates from raw chicken meat revealed that most of the isolates were resistant to amoxycillin (95.7%) followed by enrofloxacin (60.9%) and colistin (39.1%). These results show that the *E*. *coli* isolated from poultry meat sold in local markets are multidrug-resistant. Although some of the isolates appeared to be susceptible to colistin upon disc diffusion test, the same isolates were found to be positive for colistin resistance encoding gene, *mcr*-1. This discrepancy might be attributed to the less reliability of AST determination by disc diffusion test as compared to PCR which is the gold standard for detection of antimicrobial resistant bacteria [18]. It has also been reported that disc diffusion, although it is simple and easy to perform and interpret, is less reliable as compared to agar dilution and determination of minimum inhibitory concentration [19–21]. The emergence of antibiotics resistance in bacteria may occur as a result of horizontal transmission of resistance genes and transmissible elements like plasmids among bacterial species and strains [22]. Moreover, different species of bacteria are able to pass their resistance genes to their offspring by replication or to related bacteria through conjugation [23]. In *E*. *coli*, acquisition of resistance genes occurs through exchanges of resistance genes through conjugation [24]. It has also been reported that multiple antibiotic resistance may be acquired through mobile genetic elements such as plasmids, transposons, and class 1 integrons [25].

Meanwhile, PCR detection results confirmed that 29/31 (93.55%) of phenotypically identified isolates were confirmed to be *E*. *coli*. This difference between PCR and routine bacteriology results shows that molecular methods are superior to phenotypical detection for the identification of bacterial species. As for the detection of colistin resistance, 41.38% (12/29) *E*. *coli* isolates from raw chicken meat showed positive results for resistance gene, *mcr*-1. This finding confirms the presence of colistin resistance in local *E*. *coli* isolates from raw chicken meat sold at local markets. The results may also imply that the colistin-resistant *E*. *coli* might have originated from the source, i.e., poultry farms, and the possible emergence and spread of colistin resistance in poultry farms and beyond. The detection rate recorded in the current study is higher compared to other similar studies conducted elsewhere. A similar study conducted in Brazil, South America, reported lower detection rate (19.5%) of colistin-resistant *E*. *coli* harbouring *mcr*-1 genes in chicken meat samples collected from local markets in Sao Paulo [26]. Another study from Pakistan conducted on screening of 100 healthy broiler chicken reported a much lower prevalence of 8% (8/100) colistin-resistant *E*. *coli* [27]. The emergence and spread of colistin-resistant *E*. *coli* in farm animals and animal products such as chicken meat raises a serious public health concern that needs to be addressed with a sense of urgency. Colistin being a last-resort antibiotic for the treatment of infections in humans caused by Gram-negative bacteria like *E*. *coli*, the occurrence and spread of colistin resistance in animals and its detection in food is concerning. In this study, the resistance gene, *mcr*-2, was not detected. In conclusion, it is very concerning and is an alarming issue that colistin resistance is emerging and spreading in food animals and their products. The current findings also revealed that colistin-resistant *E*. *coli* are prevalent in chicken meat intended for human consumption. These findings underscore the importance and urgency to counter the emergence and spread of these resistant bacteria. Further comprehensive studies including molecular typing, virulence genes profiling, and tracing of potential sources of colistin-resistant *E*. *coli* are recommended.

## Figures and Tables

**Table 1:** Antimicrobial susceptibility patterns of *E*. *coli* (*n* = 29) isolates from raw chicken meat and bean sprouts.

Antibiotic disc (concentration, *µ*g)	Types of samples					
	Raw chicken meat (*n* = 23)			Bean sprouts (*n* = 6)		
	*R*	*I*	*S*	*R*	*I*	*S*
	No. (%)	No. (%)	No. (%)	No. (%)	No. (%)	No. (%)
AML (10 *µ*g)	22 (95.7)	0 (0)	1 (4.3)	2 (33.3)	3 (50.0)	1 (16.7)
CT (10 *µ*g)	9 (39.1)	—	14 (60.9)	1 (16.7)	—	5 (83.3)
ENR (5 *µ*g)	14 (60.9)	7 (30.4)	2 (8.7)	0	0 (0)	6 (100)
CN (10 *µ*g)	7 (30.4)	1 (4.3)	15 (65.2)	0 (0)	0 (0)	6 (100)

AML: amoxycillin/clavulanic acid, ENR: enrofloxacin, CN: gentamicin, and CT: colistin.

**Table 2:** Multidrug-resistant (MDR) isolates of *E*. *coli* from raw chicken meat and bean sprouts.

Antibiotic drugs as MDR	Source of MDR isolates	
	Raw chicken meat no. (%)	Bean sprouts no. (%)
AML, ENR	14 (26.4)	0 (0)
AML, CT	9 (17.0)	0 (0)
AML, CN	8 (15.1)	0 (0)
AML, ENR, CT	7 (13.2)	0 (0)
AML, ENR, CN	6 (11.3)	0 (0)
AML, CT, CN	5 (9.4)	0 (0)
AML, ENR, CT, CN	4 (7.5)	0 (0)
Total	53	0

AML: amoxycillin/clavulanic acid, ENR: enrofloxacin, CN: gentamicin, and CT: colistin.

## Data Availability

All the required data are included in the manuscript.

## References

[1] Huttner A., Harbarth S., Carlet J. (2013). Antimicrobial resistance: a global view from the 2013 world healthcare-associated infections forum. *Antimicrobial Resistance and Infection Control*.

[2] Akond M. A., Alam S., Hasan S. M. R., Mubassara S., Uddin S. N., Shirin M. (2009). Bacterial contaminants in carbonated soft drinks sold in Bangladesh markets. *International Journal of Food Microbiology*.

[3] Cantón R., Morosini M.-I. (2011). Emergence and spread of antibiotic resistance following exposure to antibiotics. *FEMS Microbiology Reviews*.

[4] Economou V., Gousia P. (2015). Agriculture and food animals as a source of antimicrobial-resistant bacteria. *Infection and Drug Resistance*.

[5] Altekruse S. F. (1997). Emerging foodborne diseases. *Emerging Infectious Diseases*.

[6] Biswas A. K., Kondaiah N., Anjaneyulu A. S. R., Mandal P. K. (2011). Causes, concerns, consequences and control of microbial contaminants in meat-A review. *International Journal of Meat Science*.

[7] Falagas M. E., Kasiakou S. K., Saravolatz L. D. (2005). Colistin: the revival of polymyxins for the management of multidrug-resistant gram-negative bacterial infections. *Clinical Infectious Diseases*.

[8] Rebelo A. R., Bortolaia V., Kjeldgaard J. S. (2018). Multiplex PCR for detection of plasmid-mediated colistin resistance determinants, *mcr*-1, *mcr*-2, *mcr*-3, *mcr*-4 and *mcr*-5 for surveillance purposes. *Euro Surveillance*.

[9] Frederick A. (2011). Escherichia coli, it prevalence and antibiotic resistant in Malaysia: a mini review. *Microbiology Journal*.

[10] Clinical and Laboratory Standards Institute (CLSI), Performance Standards for Antimicrobial Susceptibility Testing, Twenty-Third Informational Supplement 2013, M100-S23

[11] Elnahriry S. S., Khalifa H. O., Soliman A. M. (2016). Emergence of plasmid-mediated colistin resistance genemcr-1in a clinical *Escherichia coli* isolate from Egypt. *Antimicrobial Agents and Chemotherapy*.

[12] Liassine N., Assouvie L., Descombes M.-C. (2016). Very low prevalence of *mcr*-1/*mcr*-2 plasmid-mediated colistin resistance in urinary tract Enterobacteriaceae in Switzerland. *International Journal of Infectious Diseases*.

[13] Kong R. Y. C., So C. L., Law W. F., Wu R. S. S. (1999). A sensitive and versatile multiplex PCR system for the rapid detection of enterotoxigenic (ETEC), enterohaemorrhagic (EHEC) and enteropathogenic (EPEC) strains of *Escherichia coli*. *Marine Pollution Bulletin*.

[14] Rouger A., Tresse O., Zagorec M. (2017). Bacterial contaminants of poultry meat: sources, species, and dynamics. *Microorganisms*.

[15] Rasheed M. U., Thajuddin N., Ahamed P., Teklemariam Z., Jamil K. (2014). Antimicrobial drug resistance in strains of *Escherichia coli* isolated from food sources. *Revista do Instituto de Medicina Tropical de São Paulo*.

[16] Mritunjay S. K., Kumar V. (2017). A study on prevalence of microbial contamination on the surface of raw salad vegetables. *3 Biotech*.

[17] Steele M., Odumeru J. (2004). Irrigation water as source of foodborne pathogens on fruit and vegetables. *Journal of Food Protection*.

[18] Reller L. B., Weinstein M., Jorgensen J. H., Ferraro M. J. (2009). Antimicrobial susceptibility testing: a review of general principles and contemporary practices. *Clinical Infectious Diseases*.

[19] Taormina P. J., Beuchat L. R., Slutsker L. (1999). Infections associated with eating seed sprouts: an international concern. *Emerging Infectious Diseases*.

[20] Khalili H., Soltani R., Negahban S., Abdollahi A., Gholami K. (2012). Reliability of disk diffusion test results for the antimicrobial susceptibility testing of nosocomial grampositive microorganisms: is E-test method better?. *IJPR*.

[21] Lehtopolku M., Kotilainen P., Puukka P. (2012). Inaccuracy of the disk diffusion method compared with the agar dilution method for susceptibility testing of *Campylobacter* spp. *Journal of Clinical Microbiology*.

[22] Schroeder M., Brooks B., Brooks A. (2017). The complex relationship between virulence and antibiotic resistance. *Genes*.

[23] von Wintersdorff C. J. H., Penders J., van Niekerk J. M. (2016). Dissemination of antimicrobial resistance in microbial ecosystems through horizontal gene transfer. *Frontiers in Microbiology*.

[24] Domingues S., Nielsen K. M., da Silva G. J. (2012). Various pathways leading to the acquisition of antibiotic resistance by natural transformation. *Mobile Genetic Elements*.

[25] Partridge S. R., Kwong S. M., Firth N., Jensen S. O. (2018). Mobile genetic elements associated with antimicrobial resistance. *Clinical Microbiology Reviews*.

[26] Monte D. F., Mem A., Fernandes M. R. (2017). Chicken meat as a reservoir of colistin-resistant *Escherichia coli* strains carrying mcr-1 genes in South America. *Antimicrobial Agents and Chemotherapy*.

[27] Lv J., Mohsin M., Lei S (2018). Discovery of a mcr-1-bearing plasmid in commensal colistin-resistant *Escherichia coli* from healthy broilers in Faisalabad, Pakistan. *Virulence*.

